# Global distribution of modern shallow-water marine carbonate factories: a spatial model based on environmental parameters

**DOI:** 10.1038/s41598-019-52821-2

**Published:** 2019-11-11

**Authors:** Marie Laugié, Julien Michel, Alexandre Pohl, Emmanuelle Poli, Jean Borgomano

**Affiliations:** 1 Aix Marseille Univ, CNRS, IRD, INRA, Coll. France, CEREGE, Aix-en-Provence, France; 2 MODIS Pau, 4 Rue Jules Ferry, Pau, France; 3 Total CSTJF, Avenue Larribeau, 64000 Pau, France

**Keywords:** Biogeography, Sedimentology

## Abstract

Prediction of carbonate distributions at a global scale through geological time represents a challenging scientific issue, which is critical for carbonate reservoir studies and the understanding of past and future climate changes. Such prediction is even more challenging because no numerical spatial model allows for the prediction of shallow-water marine carbonates in the Modern. This study proposes to fill this gap by providing for the first time a global quantitative model based on the identification of carbonate factories and associated environmental affinities. The relationships among the four carbonate factories, i.e., “biochemical”, “photozoan-T”, “photo-C” and “heterozoan-C” factories, and sea-surface oceanographic parameters (i.e., temperature, salinity and marine primary productivity) is first studied using spatial analysis. The sea-surface temperature seasonality is shown to be the dominant steering parameter discriminating the carbonate factories. Then, spatial analysis is used to calibrate different carbonate factory functions that predict oceanic zones favorable to specific carbonate factories. Our model allows the mapping of the global distribution of modern carbonate factories with an 82% accuracy. This modeling framework represents a powerful tool that can be adapted and coupled to general circulation models to predict the spatial distribution of past and future shallow-water marine carbonates.

## Introduction

Carbonate systems are a major component of the Earth System. Hosting more than 25% of the marine life^1^, carbonates also represent major reservoir rocks for water and hydrocarbon resources. Moreover, carbonate rocks constitute a major carbon sink as they account for 25% of the global CO_2_ sink into marine sediments^2^. Improving our global knowledge of marine carbonate systems is therefore critical for the study of the global carbon cycle^3^ and more generally for the understanding of past and future climate changes.

Modern and ancient marine carbonates are well developed from tropical waters to polar regions, under various environmental conditions and away from large terrigenous input^4–7^. These carbonate sediments show countless biotic associations, sedimentary facies and stratigraphic architectures^8–10^. Various carbonate classifications have been proposed on the basis of grain associations^8,11^ and key controlling parameters such as tectonic settings^12^, hydrodynamics^13,14^, oceanographic parameters^11,15–21^ or biota and nutrients^10,22,23^. Thus, marine carbonate platforms are not randomly distributed in the world oceans, neither in the Modern, nor in the Past^9,24^. Global mapping of carbonate platforms shows indeed that simple oceanography-based trends do exist at a global scale^9,15,24–27^. These trends between carbonate occurrences and oceanography are well integrated in the carbonate classifications of James into photozoan and heterozoan grain associations^7,8^ and of Schlager into tropical shallow-water (“T”), cool-water (“C”) and mud-mound (“M”) carbonate factories^9,16,17^; a factory being defined as a carbonate precipitation mode that is characterized by an ecosystem. Continuing these classifications and following the Schlager approach^9^, Michel *et al*.^28^ further defined four platform-scale carbonate factories that are the “heterozoan-C”, “photo-C”, “photozoan-T” and “biochemical” factories.

Despite these conceptual classifications and apart from the temperature- and salinity-based diagrams of Lees (1975)^15^, no numerical spatial model accounting for all types of shallow-water marine carbonates allows for the prediction of carbonate locations at a global scale. Some models simulate carbonate production rates in the modern ocean^2,29–31^, but only consider coral reefs. The model of Martin *et al*.^32^ aims to predict the location of carbonates into the Mediterranean Sea, but only considers coralligenous and maërl sediments. The ReefHab model of Kleypas *et al*.^30^ provides a fine estimate of the global distribution of coral reef systems in both the Modern and the Past, but does not consider other types of carbonates. The carbonate factory function of Pohl *et al*.^27^ predicts the occurrences of the Cretaceous tropical shallow-water carbonate factory at a global scale based on modelled paleoceanographic parameters (sea-surface temperature - SST, sea-surface salinity - SSS, marine primary productivity) and water depth, but it does not make any distinction between the different types of carbonate associations and does not consider cool-water carbonates. The lack of a comprehensive numerical spatial model of modern carbonates at a global scale, including diverse shallow-water marine carbonate factories, poses this critical question: how can we envisage and trust the prediction of ancient carbonates at a global scale if there is no robust model in the Modern?

The aim of this study is therefore to propose a global predictive model, accounting for diverse modern shallow-water marine carbonates. The carbonate factory classification of Michel *et al*.^28^ that was first defined for a rock record perspective is used in the present study. Our model uses carbonate factory functions^27^ to derive carbonate factory occurrences from key oceanographic parameters: SST, SSS, marine primary productivity and water depth^22^. Other types of carbonates, including continental carbonates, deep-water coral mounds, carbonate seeps and pelagic carbonates, are not considered here.

## Results

### General approach

The *a priori* classification of modern carbonate platform data into a carbonate factory scheme is deterministic. This classification follows the approach of Schlager^9^ and Michel *et al*.^28^ and is based on basic ecological and sedimentological knowledge about carbonate-producing biota and grains^8–10,22,23^. A single factory is assigned to the whole platform, depending on dominant characteristic carbonate producers, that might not be exclusive to a given factory. Other secondary carbonate-producers can also occur in the platform, and are included into the main factory.

**Figure 1:**
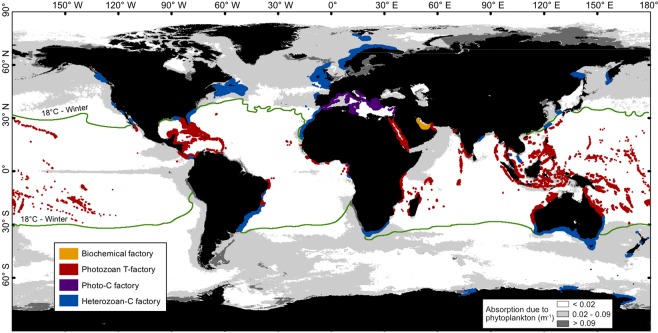
Observed worldwide distribution of modern shallow-water marine carbonate factories. Carbonate factory extent is exaggerated for visualization purpose. Background oceanographic map corresponds to remote-sensing data of marine primary productivity (absorption due to phytoplankton). Carbonate platform distribution come from bibliographic data (see text for details). The 4 factories are defined after Michel *et al*.^22,28^, by splitting the Schlager^9,16,17^ T-factory into “biochemical” and “photozoan-T” factories and the C-factory into “photo-C” and “heterozoan-C” factories.

The relationship between oceanographic parameters and carbonate factories is studied using spatial analysis for both winter and summer seasons. Maps of SST, SSS and marine primary productivity are extracted from remote-sensing data (see Methods). The distribution of modern carbonate factories is mapped based on bibliographic data (Fig. 1), a single factory being assigned to the whole platform. The spatial analysis method is similar to the one used by Kleypas *et al*.^26,30^ that compares oceanographic parameters to the coral reef distribution (our “photozoan-T factory” plus the specific corals of the Persian Gulf), with the difference that the present study includes all shallow-water marine carbonates and is performed seasonally (summer and winter) for each parameter. The results of the statistical analysis form the basis for building carbonate factory functions following the method of Pohl *et al*.^27^. Functions simulate a spatial distribution of carbonate factories from environmental parameter maps. The carbonate factory functions predict neither biota nor sediment types; they identify regions showing appropriate environmental conditions (i.e., extrinsic parameters) for different types of carbonate production to occur. The complete workflow (see Supplementary Fig. S1), i.e., mapping, data processing, spatial statistics and modeling, is realized using the Environmental Systems Research Institute (ESRI) software ArcGIS® (v10.2.2).

### Spatial analysis and carbonate factory definition

**Figure 2:**
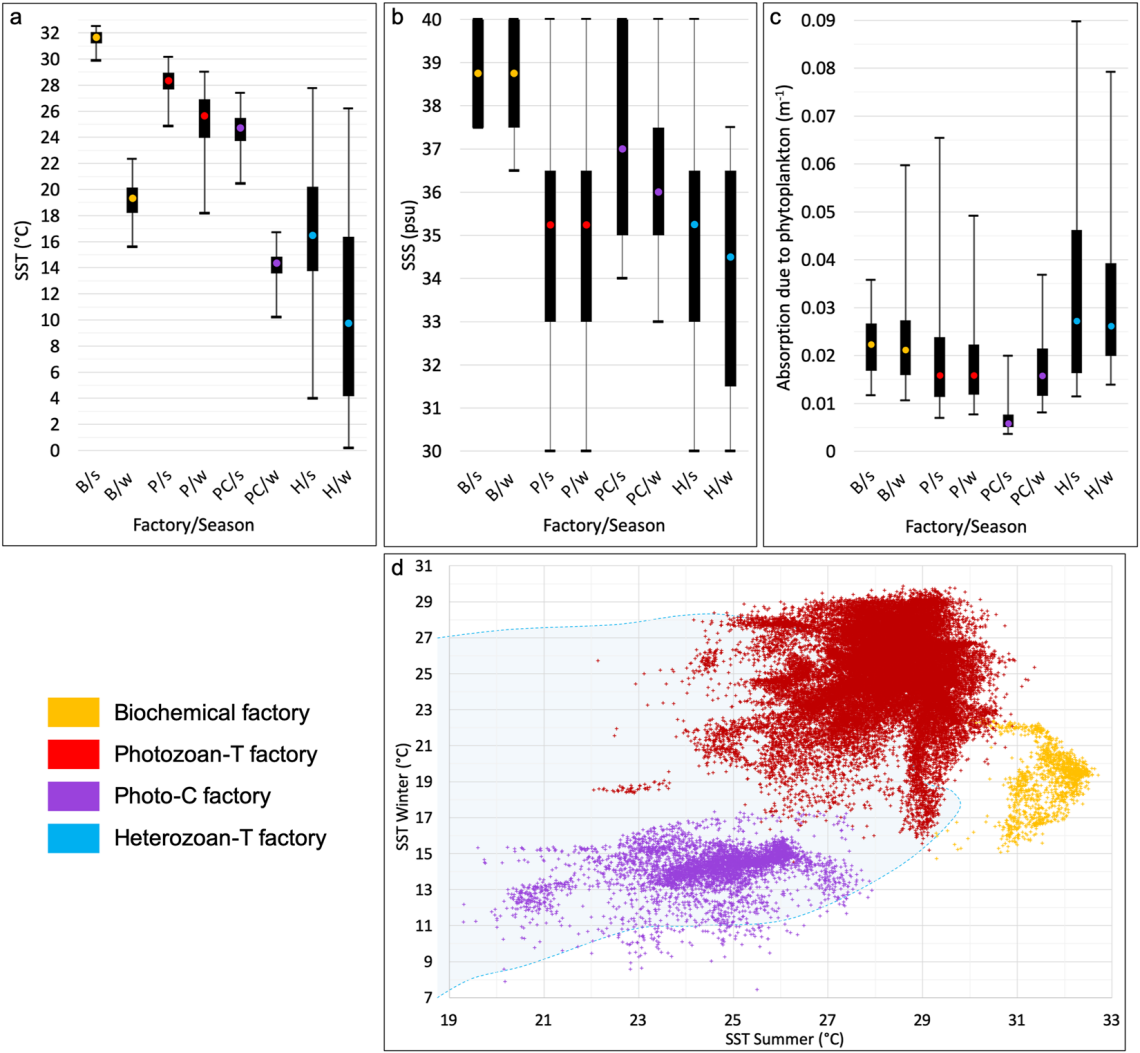
Results of the spatial analysis. (**a–c**) Box-and-whisker plots obtained from the spatial analysis for each factory/oceanographic parameter/season (B - biochemical factory, P - photozoan-T factory, PC - photo-C factory, H - heterozoan-C factory, s - summer, w - winter). Whiskers respectively correspond to the 1st and 99th percentiles for SST and SSS and to the 5th and 95th percentiles for the absorption due to phytoplankton; box extremities show the 25th and 75th percentiles; colored points display the median value. (**d**) SST summer/winter cross-plot obtained from the spatial analysis showing discriminative temperature ranges for the biochemical factory, photozoan-T factory and photo-C factory. Heterozoan-C factory dots are not represented as this factory is found under a large temperature range (blue shaded area). Salinity ranges are very large for all of the factories, except for the biochemical factory. The latter is characterized by a very restricted range of salinity, which appears as a key parameter for this factory. Marine productivity value ranges are very large and similar for each factory. For this reason, marine productivity does not allow discriminating the different factories. Nevertheless, it is an important parameter that controls the occurrence and extent of factories in upwelling areas.

The spatial analysis shows that the four groups of shallow-water factories effectively correspond to specific environmental conditions (Fig. 2a–c). Sea-surface temperature seasonality is the main discriminative parameter that allows for the exclusive numerical distinction of the biochemical, photozoan-T and photo-C factories (Fig. 2d). Marine productivity also plays an important role by controlling the occurrence of carbonate factories. Nevertheless, marine productivity has similar value ranges (Fig. 2c) for the different carbonate factories and thus do not allow defining the factories numerically. Finally, salinity plays a major role only for the biochemical factory.

#### The “biochemical factory”: very warm and salty waters

The area corresponding to the Persian Gulf shows the warmest SST with summer temperatures above 30 °C (Fig. 2a). This is the only marine location on Earth reaching such high temperatures. SSS are very high with values above 37.5 psu all year round (Fig. 2b). Oceanic primary productivity is moderate and non-discriminative as suggested by the large range of productivity values found in the Gulf (0.01 to 0.06 m^−1^ phytoplankton absorption; mean of 0.02 m^−1^; Fig. 2c).

The Persian Gulf which is an extensive, slightly dipping ramp setting represents a unique tropical carbonate platform environment in the modern world. Carbonate sediments of the Persian Gulf ramp include non-skeletal grain deposits such as oolite shoals, microbial deposits such as stromatolites or other algal mats, foraminifera and bivalve sediments^33,34^, and evaporitic deposits such as sabkhas. Some specific corals are also present but they are sparse and extreme temperatures during both winter and summer seasons are responsible for high mortality rates, a low diversity, and a relatively deep habitat^33,35,36^. Corals do not build large structures and do not modify the geometry of the sedimentary profile^37^. They are thus secondary carbonate producers in the Persian Gulf.

Characteristic deposits of this factory correspond to a chemically-driven precipitation mode, which is consistent with a hot and salty marine environment^22,28,38,39^. As highlighted by Michel *et al*.^22,28^, this carbonate association occurs in the most carbonate-saturated waters. SST and SSS are thus the major factors controlling the deposition of the biochemical factory. The pronounced seasonal SST change does not appear to affect the nature of the sedimentary environment. By contrast with other photozoan grain associations, such as those dominated by coral reef rimmed platforms^28^, SST of the Persian Gulf decrease down to 15 °C during winter without inhibiting carbonate sedimentation.

#### The photozoan-T factory: tropical zone

The tropical zone is characterized by SSTs that are warm and relatively constant year-around, ranging from 18 °C to 30.5 °C (Fig. 2a). Oceanic productivity is relatively low (averaging at 0.02 m^−1^ phytoplankton absorption; Fig. 2c). A wide range of SSS values is recorded for this carbonate factory (from 30 to 40 psu – Fig. 2b).

SST appears to be the major factor controlling the global distribution of the photozoan-T factory. As demonstrated by Kleypas *et al*.^26^, the yearly temperature minimum of 18 °C is a critical threshold as the factory does not occur beyond this isotherm. Furthermore, spatial analysis reveals SST has to rise above 24 °C during summer to permit the development of the photozoan-T factory. During winter, the photozoan-T factory persists if SST drops below 24 °C but it disappears if the mean SST drops below 18 °C. Areas where SST remains above 18 °C but never exceeds 24 °C do not host the photozoan-T factory (e.g., the southwestern corner of Western Australia). Two critical thresholds are thus identified: (i) 24 °C as the minimum SST for the carbonate factory production and (ii) 18 °C as the minimum SST for the carbonate factory persistence (see Supplementary Fig. S2). The 30.5 °C threshold corresponds to the maximum SST that the photozoan-T factory can withstand. These carbonates correspond to the typical coral reef platform systems^4,5,28^ that include secondary producers such as non-skeletal, foraminifera and green and red algae, also known as photozoan carbonates^8^, the T-factory^9^ or warm-water carbonates^7^. Another controlling parameter is the marine productivity, which must remain moderate not to inhibit the photozoan-T factory, as observed in upwelling areas along the western margins of the American and African continents. Temperature thus stands out as the main controlling factor along the latitudinal axis, while marine productivity controls the east-west trend in global carbonate platform distribution.

#### The photo-C factory: warm-temperate province

The warm-temperate province corresponds to mid-latitude zones where SST shows either a strong seasonality ranging from 10 °C to 28 °C (cold winter, warm summer) or intermediate temperatures all year round between 18 °C and 24 °C (Fig. 2a). These climatic zones include the Mediterranean Sea, part of the southern Australian coast (western Great Australian Bight), the Japanese coasts and California. A comparison with the Köppen-Geiger climate classification map^40,41^ reveals that these photo-C factory zones mainly correspond to the Mediterranean climate (climates Csa and Csb of the Köppen-Geiger classification). Oceanic productivity is moderate (<0.04 m^−1^ phytoplankton absorption; Fig. 2c) and SSS is relatively high (>33 psu; Fig. 2b).

Carbonates of the photo-C-factory include widespread red algal deposits and seagrass-derived sediments such as foraminifera grains and are also known as the Mediterranean platform scheme of Pérès and Picard (1964)^42^, the microtidal cool-water carbonates^43^, and the protected-setting type of the warm-temperate carbonates^7,21^. By contrast to the photozoan-T factory, in which tropical seagrass and red algal depositional environments occur^28^, the photo-C-factory does not show any significant coral reef systems, probably due to inappropriate temperature ranges^26^.

**Figure 3:**
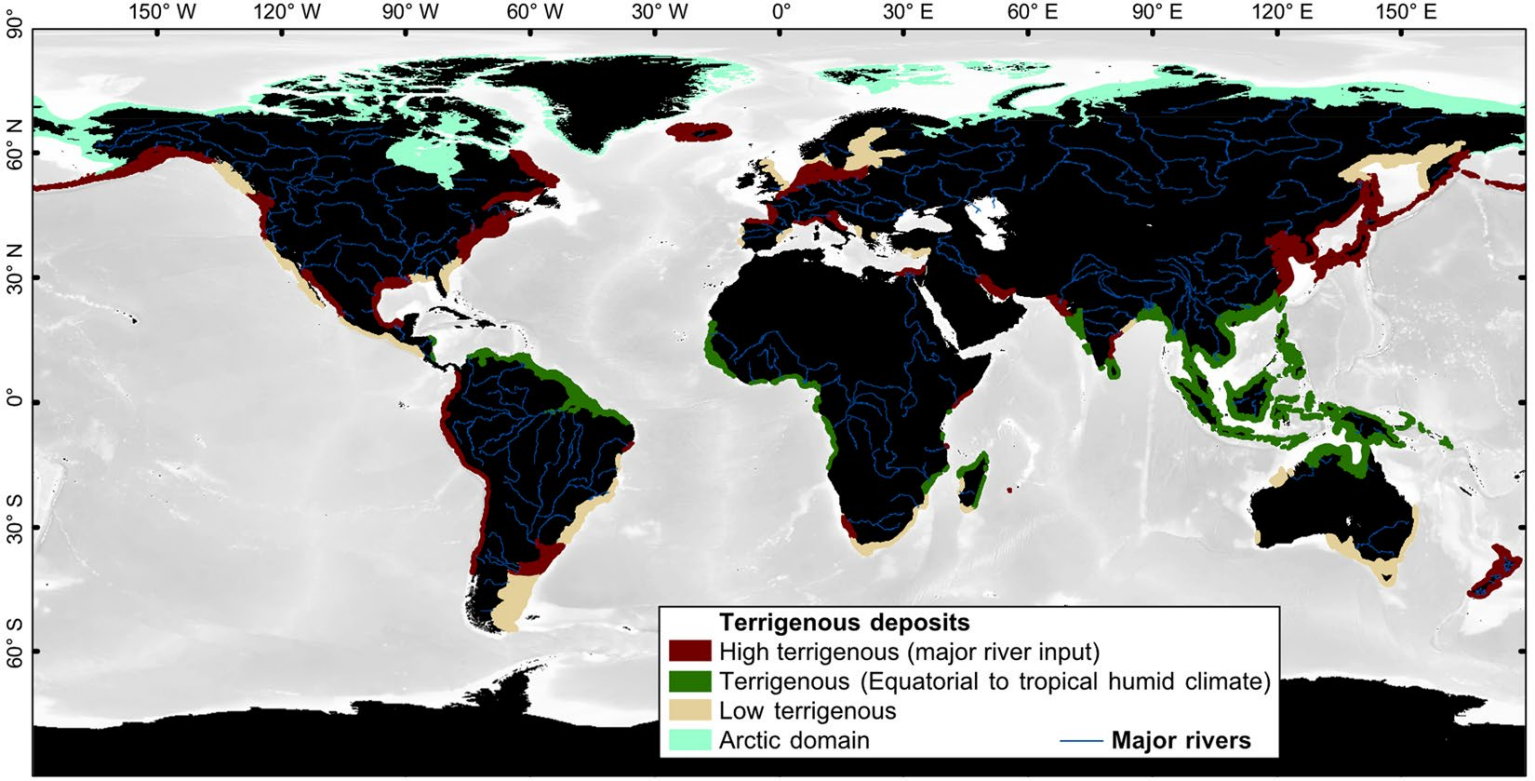
Shelfal distribution of terrigenous deposits. Deposits are classified into 4 categories: highly terrigenous deposits (from major river input), terrigenous deposits under humid climatic conditions, low terrigenous input (small rivers and/or drainage basins) and terrigenous deposits related to the Arctic domain. “High terrigenous”, “Terrigenous” and “Arctic domain” inhibit (i.e., mask every carbonate factory. “Low terrigenous” class is also used as a mask, except for the heterozoan-C factory that can handle such conditions.

The need of light penetration and relatively warm temperature represents the dominant steering environmental factors controlling the production of the photo-C factory, although this factory can tolerate relatively cold conditions during winter (down to 10 °C). SST seasonality is very specific to this factory and is the major point discriminating this factory from the photozoan-T factory. Even though they are within the SST range mentioned above, areas such as SW and NW Africa and America, SE Australia, eastern Asia and Japan, eastern North America, Uruguay and Argentina do not show the photo-C factory because they correspond to either zones of higher oceanic productivity (i.e., upwelling) or to areas under the influence of terrigenous input (Fig. 3).

#### The heterozoan-C factory: the “background” factory

These carbonates are found under variable environmental conditions with SST ranging from 0 to 28 °C (Fig. 2a). No specific SST values could be determined for this carbonate factory. SSS also shows a large range of values from 30 to 40 psu (Fig. 2b). Oceanic productivity ranges from low to high values (0.01‒0.09 m^−1^ phytoplankton absorption; Fig. 2c). This factory includes widespread bryozoan and bivalve deposits and is also known as cool-water carbonates, or heterozoan carbonates (James, 1997)^8^, but excluding carbonates of the photo-C factory as described in Michel *et al*.^22,28^.

Primary productivity is the dominant steering oceanographic parameter controlling the development of the heterozoan-C factory. Indeed, the highest values of primary productivity are specific to the heterozoan-C factory (Fig. 2c). This pattern is consistent with a high oceanic productivity that favors the development of suspension-feeding biota^22,23,44,45^. Nevertheless, the wide range of oceanic productivity values associated with heterozoan-C factory occurrences do not permit to discriminate this factory from the others. Even though the heterozoan-C factory contributes to the only carbonate production occurring in cold waters (i.e., <10 °C), SST and SSS do not constitute key exclusive parameters of the heterozoan-C factory that occurs over a large range of values.

### From environmental parameters to the carbonate factory functions

Based on the results of the statistical analyses (cf. “Results ”, and Table 1), carbonate factory functions were determined for each carbonate factory [Eq. (1) and Table 1] following the method developed by Pohl *et al*.^27^.

1 $${\rm{F}}({\rm{c}}{\rm{a}}{\rm{r}}{\rm{b}})=[{\rm{a}}\,{\rm{f}}(z)+{\rm{b}}\,{\rm{f}}(SST)+{\rm{c}}\,{\rm{f}}(SSS)+{\rm{d}}\,{\rm{f}}(P)]-[{\rm{t}}{\rm{e}}{\rm{r}}{\rm{r}}]$$

where: *z* – Bathymetry; *SST* - Sea-surface temperature; *SSS* - Sea-surface salinity; *P* - Oceanic productivity (absorption due to phytoplankton); terr - Terrigenous deposits; f(*x*) - Susceptibility of occurrence for each environmental parameter *x*; a + b + c + d = 1 - inter-parameter weighting factors; F(carb) - Susceptibility of occurrence of the considered carbonate factory.

**Table 1:** Types of functions and associated mathematical parameters, inter-parameter weighting factors and susceptibility thresholds for each carbonate factory.

		Biochemical factory	Photozoan-T factory	Photo-C factory		Heterozoan-C factory
Salinity	Type of function	“Near Gaussian” [Eq. (3)]	“Gaussian” [Eq. (2)]	“Gaussian” [Eq. (2)]		Independent
	Mid-Point	8 (>37.5 psu)	5 (35–35.5 psu)	7 (36.5–37.5 psu)		
	Spread	3	0.043	0.075		
	Weighting factor	0.45	0.06	0.12		0
Productivity	Type of function	Independent	“Gaussian” [Eq. (2)]	“Gaussian” [Eq. (2)]		“Gaussian Near” ([Eq. (3)]
	Mid-Point		0.015	0.0203		0.06
	Spread		565	2450		625
	Weighting factor	0	0.27	0.22		0.9
Temperature	Type of function	“Sigmoid – Large” [Eq. (4)]	“Gaussian” [Eq. (2)]	“Gaussian” (Production) [Eq. (2)]	“Gaussian” (Persistence) [Eq. (2)]	Independent
	Mid-Point	30.5	25.7	25.25	12.325	
	Spread	58	0.03	0.025	0.0244	
	Weighting factor	0.45	0.64	0.62		0
Final threshold values		0.6	0.7	0.5		0.5

Salinity values are reclassified into classes numbered from 1 to 8 (see Methods and Data section). The MP value for the Photozoan-T factory is calculated by considering the two threshold values 24.15 °C (production) and 17.65 °C (persistence): MP - (Threshold Min + Threshold Max)/2, with Max - 30.5 °C and Min - (24.15 + 17.65)/2. These values are obtained from the statistical analysis and correspond to summer and winter 1^st^ percentile, respectively. To obtain the f(*SST*) function for the Photo-C factory, the persistence and production functions are multiplied to keep the seasonality effect.

**Figure 4:**
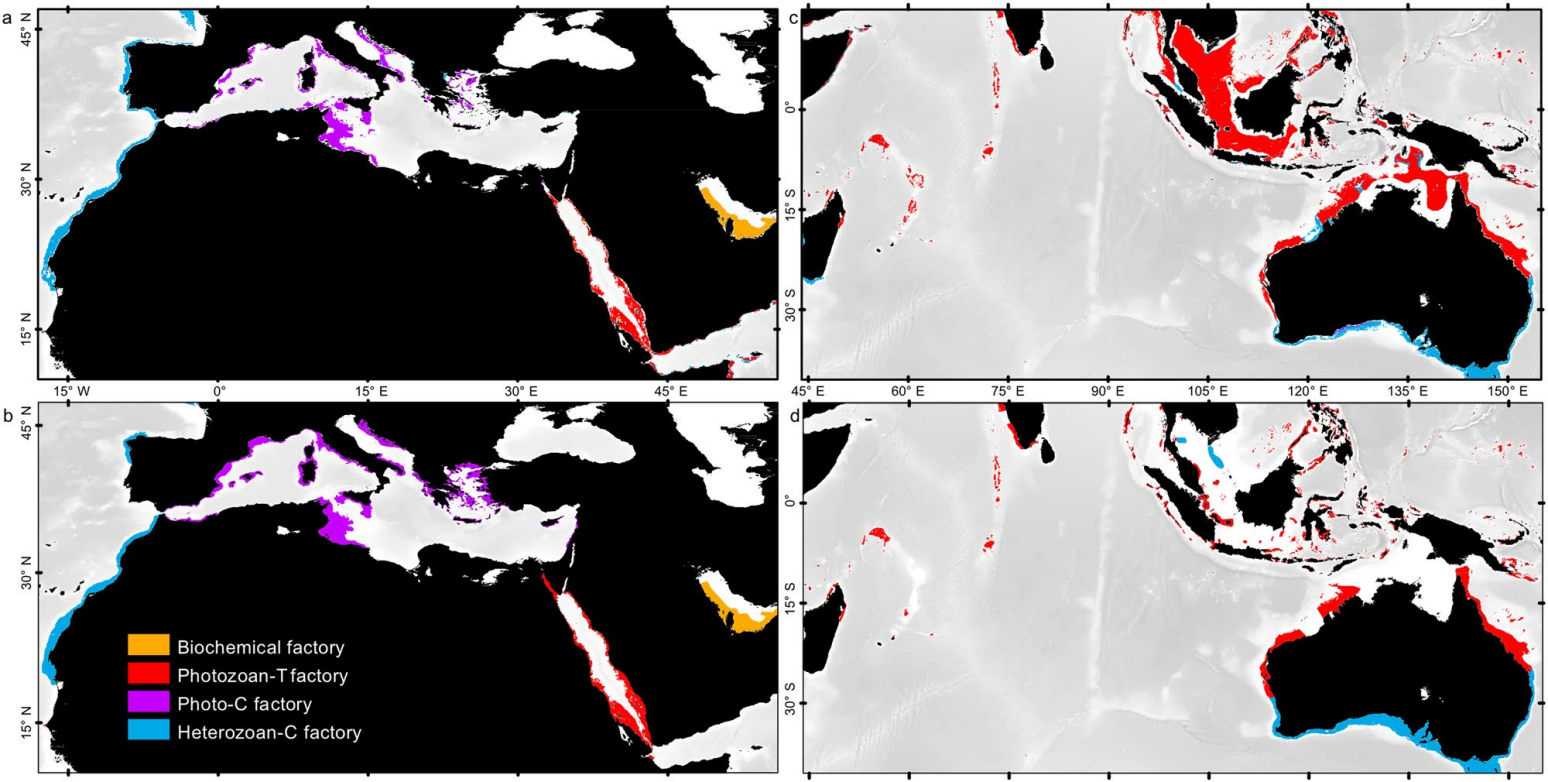
Simulated modern carbonate factory distribution and comparison with the observation map. (**a**) Close-up of the simulated map around the Mediterranean Sea and (**b**) corresponding observation map based on bibliographic data (zoomed from Fig. 1). General distributional trends of each carbonate factory are well reproduced. Modeling anomalies are observed at a local scale (see Fig. 5). (**c**) Indian ocean-centered close-up of the simulated map and (**d**) corresponding observation map based on bibliographic data (zoomed from Fig. 1). General trends of the heterozoan-C and photozoan-T factories are well reproduced, but major anomalies are found on the large epicontinental Sunda and South China terrigenous-influenced shelves (see Fig. 5). See Fig. S3 for the global map of simulated carbonate cover.

These functions are applied in every shallow-water grid point away from terrigenous fluxes (i.e., excluding the zones covered by the bathymetric and terrigenous masks; Fig. 3). Four distribution maps (i.e., one map for each carbonate factory) are then obtained that show the predicted susceptibilities of occurrence of the carbonate factories with values ranging between 0 (i.e., no susceptibility of occurrence) and 1 (i.e., maximum susceptibility of occurrence). For each calculated map, a threshold value is determined (Table 1) by comparing the model output with the observed distribution of modern carbonates. In the model, a carbonate factory develops in regions where its simulated susceptibility of occurrence is higher than the threshold value defined for the factory. This step permits maps of susceptibility of occurrence to be converted into binary maps of predicted presence/absence. The four resulting maps of occurrence are then combined into a final modelled map of distribution of the different carbonate factories showing in each point of the Earth the carbonate factory that is expected to develop (if any – Fig. 4a,c).

### Simulated map of the global distribution of modern carbonate factories

**Figure 5:**
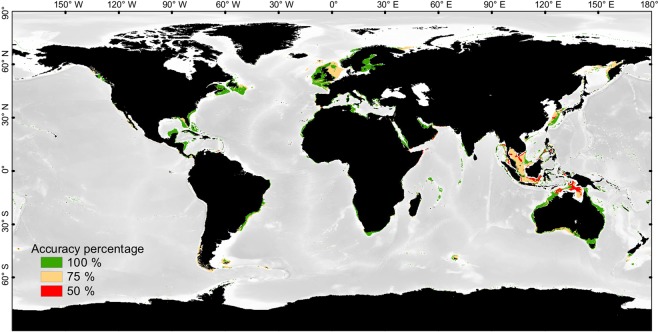
Modeling accuracy map showing the differences between the observation map of shallow-water carbonate distribution and the simulated map obtained using the carbonate factory functions. The 4 carbonate factories are simulated in each cell of the grid, as absent or present. An agreement with the observation map is counted as a success, a misfit as a fail. Accuracy in each cell can range between 100% if the four factories are correctly estimated (absence or presence) to 0% if the four factories are wrongly estimated. An accuracy of 75% means that 3 out of the 4 factories are correctly estimated. Most anomalies are related to uncertainties in the distribution of terrigenous deposits on large epicontinental shelves (see Fig. 4c,d).

The simulated map of carbonate factories (Fig. 4a,c; see also Supplementary Fig. S3) reproduces the observed distribution of carbonate factories (Fig. 4b,d) with 82% global accuracy (Fig. 5). The global distributions of the carbonate factories do not overlap at a global scale; each one occurs in well-defined, exclusive areas corresponding to specific environmental conditions, apart from the heterozoan-C factory, which is a background factory, overwhelmed by other factories.

#### Modelled distribution of carbonate factories

Main distributional trends of the four carbonate factories are well reproduced by numerical modeling (Fig. 4a,b).

The warmest and highly saline waters during at least one season (SST >30.5 °C and SSS > 37.5 psu) of the Persian Gulf predicts the occurrence of the biochemical factory. These specific oceanographic conditions could explain the uniqueness of this carbonate ramp in the modern world at a global scale^33,34^. The Shark Bay and the top of Great Bahama Bank also show deposits that are characteristic of the biochemical factory^28^. These deposits, however, are restricted to the inner parts of the flat-topped platforms that show steep slope angles. At the platform scale, the northwest Australian shelves and the Bahamas are assigned to the photozoan-T factory^28^.

The photozoan-T factory is specific to tropical areas that show warm temperatures all year round (18 °C <SST < 30.5 °C), warmer temperatures for at least one season (>24 °C) and low marine productivity levels (averaging 0.02 m^−1^ phytoplankton absorption). Thus, platforms characterized by coral reef barriers and fringing coral reefs are captured by the model; from Pacific and Indian Ocean atolls to the Caribbean Sea, Red Sea and SE Asia.

Warm-temperate areas are characterized by (i) a Mediterranean climate, (ii) moderate primary productivity levels and (iii) intermediate to high salinities (>33 psu), which provide optimal conditions for the production of the photo-C factory. These environmental conditions are specific to the Mediterranean Sea in which typical warm-temperate seagrass and red algal deposits are widespread^21,28,42,43,45–47^. The western part of the Great Australian Bight also appears as favorable to the development of the photo-C factory, even though heterozoan carbonates occur in this area^48^. Further investigations of the carbonate deposits in this area reveal that rhodalgal grain associations predominates in the swell-protected western part of the Great Australian Bight, which could be consistent with the occurrence of the photo-C factory^22,49^.

The modelled heterozoan-C factory distribution is spread over wide ranges of oceanographic parameters such as SST and SSS. The only parameter that is considered for simulation is the marine primary productivity (see Table 1), the heterozoan-C factory is simulated in every area of medium to high productivity, that includes the upwelling zones and the majority of shallow-water platforms with limited terrigenous deposits (i.e., “low terrigenous” in Fig. 3). The heterozoan-C-factory could not be discriminated numerically from other factories. Hence, following the fact that filter-feeding biota that characterize the heterozoan-C-factory are r-strategist and relatively opportunistic, other factories had to be considered as overwhelming the heterozoan-C factory. This factory is thus defined as “opportunistic” or “background”. Consequently, the heterozoan-C factory only develops in areas where other factories do not occur, given a minimum marine productivity of 0.01 m^−1^ (absorption due to phytoplankton).

#### Model anomalies

The simulated map of carbonate factory distribution matches 82% of the map of carbonate occurrences (Fig. 5). The 18% anomalies mainly correspond to overestimations, that is areas where carbonates are simulated despite their absence in the database (potentially reflecting either a reality of absence of carbonates or the absence of data; Fig. 4c,d; Supplementary Table S4). Concerning the repartition between factories, 61% of the anomalies concern the heterozoan-C factory and 36% concern the photozoan-T factory. The photo-C and biochemical factories present a much higher model fidelity as they represent only 2.8% and 0.2% of the anomalies, respectively. The 18% anomalies are divided into four categories.

57% of these anomalies are related to large (hundreds of kilometers-wide) and relatively flat epicontinental shelves (Sunda, Sahul, North Sea and East China Sea shelves; Fig. 5). In those areas the model simulates carbonates all over the platforms between 0– and 100– meter water depths (set in the model as an *a priori* bathymetric range for the carbonate factory simulation). Although photozoan carbonates, represented for instance by coral reefs, do occur locally on those shelves, their distribution does not extend over the distal, low-angle zones of the shelves. The model correctly simulates the occurrence of the carbonate factories but overestimates their size basin wards. This anomaly could result from either (1) an unidentified high regional terrigenous flux that is expected under these equatorial climatic conditions, (2) a within-platform topographic effect or (3) the influence of water motion^50–52^; (2) and (3) are coupled to define the local hydrodynamic conditions over the platform that are not considered in the current, global-scale simulations. On the one hand, areas characterized by spurious overestimation are those found in tectonically active regions under the influence of a humid equatorial climate^53^ (South East Asia specifically). These specific conditions lead to a high input of terrigenous material over the region^54^, including dissolved and particulate nutrients. These terrigenous fluxes are thought to largely influence these shelves except where topographic highs are found. The “terrigenous mask” used in our modeling approach is limited to coastal settings and thus does not reach that far offshore on extensive platforms. On the other hand, the considered bathymetric interval was empirically set to 0–100 m or and 0–200 m depending on the carbonate factory of interest. These extensive continental shelves represent peculiar, terrigenous-influenced, very flat and large, shallow-water (<100 m water depth) platforms surrounding structural highs that host flourishing carbonate-producing ecosystems. A specific improvement of the model could consist in including a supplementary parameter representing the slope or the distance to a topographic high in order to constrain the lateral carbonate distribution on extensive, shallow-water platforms [similar to Martin *et al*.^32^ in their coralligenous/maërl model].

The second type of anomaly is related to bibliographic uncertainties (17%). Anomalies are calculated by comparing the modelled map with the observation map. However, the observation map is not exhaustive due to the lack of shelfal sedimentary data. In the areas where terrigenous sediments are not mapped, the model exclusively simulates heterozoan carbonates, (western North and South America, North Sea, Sea of Okhotsk). It is thus questionable whether these areas are effectively occupied by heterozoan carbonates or should be mapped as terrigenous deposits.

1% of anomalies is due to discrepancies between measured phytoplankton absorption and the location of upwelling areas. In particular, it is well known that coral reef development along the southern Oman and Somalia coasts is limited by upwelling related to the Indian southwest summer monsoon^55^. However, this upwelling is not well captured by remote-sensing data, due to sea-bottom reflection bias in areas of shallow bathymetries (shelf environment). Consequently, the model wrongly predicts the development of the photozoan-T factory in these areas.

Finally, a quarter of the anomalies could not be explained (25%). These anomalies primarily occur at the transition zone between two carbonate factories. These anomalies could thus correspond to border effects resulting from threshold-related artifacts and the environmentally exclusive definition of the carbonate factories. These anomalies include the entrance of the Persian Gulf where the photozoan-T and biochemical factories are observed side-by-side and the Great Australian Bight where both the photo-C and the heterozoan-C factories occur.

## Discussion

The proposed spatial modeling method displays an 82%-fit to the observation map of worldwide shallow-marine carbonates. Based solely on winter and summer SST, SSS and marine primary productivity, our spatial modeling method provides realistic predictions of the global distribution of the different carbonate factories in the Modern, provided that seasonality is considered. The use of a limited number of key oceanographic parameters is a prerequisite to permit the use of this method to simulate the distribution of platform carbonates in the geological past based on paleoclimatic constraints derived from general circulation models. The fairly realistic prediction of the rudist carbonate platforms of Cretaceous times (81% of occurrence prediction) demonstrates the relevance of applying this method to the geological past^27^. Thus, the three key oceanographic parameters (i.e., SST, SSS and primary productivity) are considered to provide a simple yet powerful estimation of shallow marine carbonate platforms. SST appears as a major oceanographic parameter, which largely controls biogeography and the latitudinal extent of carbonates^15,24,26^. SST alone allows for the numerical validation of three of the four carbonate factory classes, i.e., photozoan-T-factory, biochemical factory and photo-C-factory (Fig. 2). The other main oceanographic parameter, SSS, was also shown to influence the global distribution of shallow-water carbonates^15,33,56^, especially of non-skeletal and abiotic carbonates under either humid or arid climatic conditions^15,28,33,56^. Through the delivery of food for heterotrophic organisms, primary productivity is the primary energetic source for heterozoan carbonates^6,8,23,57^. It also represents a major factor controlling the occurrence of photozoan carbonates by inhibiting their development in upwelling areas^8^. Moreover, these three parameters provide a fine image of the general (paleo)climate (for instance *p*CO_2_) and (paleo)oceanographic biochemical state of the ocean. SST is correlated to solar irradiance and thus to PAR (Photosynthetically Active Radiation), an important parameter for organisms using light. SST is also correlated to the ocean carbonate saturation^26,28,58^ controlling carbonate precipitation. SSS also offers information about the ocean chemistry as it is correlated to alkalinity^59,60^, which also controls carbonate precipitation^61,62^. Finally, marine productivity is related to nutrient concentrations and oxic conditions^28^. While other parameters such as *p*CO_2_ and Mg:Ca ratio were shown to impact carbonate evolution through geological times, they were not shown to control the spatial distribution of platform carbonates at any given time^28^. This is the reason why they are not included in the carbonate factory functions. To be noted that the modeling framework is flexible and allows for the integration of parameters for future developments.

Terrigenous deposits represent a major constraint for spatial modeling of carbonate occurrences as they generally inhibit carbonate production, except for the heterozoan-C factory and marginal carbonates that can cope with relatively moderate terrigenous fluxes^22,63^. Unfortunately, terrigenous fluxes are not well constrained at a global scale. Although the mapping of terrigenous sediments is significantly facilitated in the Modern by remote-sensing and semi-quantitative data based on Milliman & Farnsworth (2011)^51^, 57% of modeling misfits are related to uncertainties in the distribution of terrigenous materials. In this study, we compiled a map of terrigenous deposits (Fig. 3), based on bibliographic and remote-sensing data, which was used as a simple mask inhibiting carbonate production (cf., Methods and Data section). Terrigenous deposits are only mapped along the coasts, which is fine for narrow continental platforms, but appears problematic for large continental shelves over which the distal extent of terrigenous deposits is poorly constrained in many cases, such as the Sunda Shelf and South China Sea. Moreover, this mapping method is difficult to apply throughout geological times. The semi-quantitative classification of terrigenous deposits could theoretically be conducted for past periods as it is based on paleogeographic and paleoclimatic data (i.e., major rivers, drainage basin areas, topography and paleoclimate). In practice however, ancient terrigenous deposits are difficult to map at a global scale because of the lack of data and geological uncertainties. A major limitation is found in very humid areas where terrigenous inputs are widespread and deposits are controlled by local topography. The distribution of carbonate and terrigenous deposits is binary and the direct interpretation of remote-sensing data is required to ensure a robust mapping. Still, a promising way forward to constrain the distribution of ancient terrigenous sediments would consist in coupling a model of continental physical erosion^64^ with a general circulation model to propose a quantification of the extent of terrigenous deposits on continental platforms.

No parameter values could be identified to discriminate the heterozoan-C factory from other factories. If low SST (down to 0 °C) and high marine productivity levels are specific to this factory, heterozoan carbonate platforms are also found in areas under high marine temperatures (up to 28 °C) and low productivities (down to 0.01 m^−1^ phytoplankton absorption). Thus, a mask was used as a mapping constraint for the distributional model of the heterozoan-C factory; this mask corresponds to the spatial distribution of other factories. Heterotrophic organisms are widespread and commonly occur in other factories (for instance on the distal parts of platforms^5^). The heterozoan-C factory is considered as opportunistic and occurs where other factories do not develop as long as terrigenous deposits are limited. This factory acts as a “background” factory, which occurs everywhere, but is overwhelmed by other factories. The spatial distribution of the heterozoan-C factory is thus controlled by the absence of other factories. Marine productivity then enhances carbonate production (under a certain limit) and is not a direct controlling parameter of heterozoan-C factory occurrence. In addition, the inhibition of the heterozoan-C factory by terrigenous deposits is not well constrained since heterotrophic organisms do thrive in terrigenous sediments. In natural environments, there is a continuum from the occurrence of a few carbonate-secreting heterotrophic organisms living in terrigenous sediments to carbonate deposits that are exclusively composed of heterotrophic biota. Thus, the transition between siliciclastic and heterozoan-C factory sediments is not well defined and which significantly impacts the robustness of our predictions for this factory. However, following simple ecological, trophic-related concepts^23^ and rock record observations^22^, significant accumulations of the heterozoan-C factory likely will only be recorded in highly productive settings^6^, or following mass extinction events that selectively eliminated most tropical carbonate producers.

By providing an 82% fit to the observation of modern carbonate factory distribution, the carbonate factory functions [cf., Eq. (1)] have demonstrated their efficiency to predict carbonate factory distribution from key oceanographic parameters, both in the Modern and in the Past by using palaeoceanographic modeling^27^. Nevertheless, the model has limitations that should be kept in mind. First, the carbonate factory concept does not provide any prediction of local biota or grain distribution within a carbonate platform as factories are considered as single carbonate production systems at the platform scale. Our model is powerful to identify global trends of carbonate production and thus to predict potential carbonate occurrences based on environmental parameters. To be able to predict biota or grain distribution and depositional profiles, it would be necessary to develop a model using local parameters (e.g., high-resolution bathymetry, hydrodynamics, 3D temperature and salinity data throughout the water column). Then, carbonate factories are carbonate precipitation modes that are defined by an ecosystem (biota and environments^9^). Because of biotic evolution, the grain composition of the carbonate factories has changed throughout the geological times and the relationship with paleoenvironmental conditions necessarily changed^65^. Carbonate factory functions defined in the Modern should not be directly applied in ancient times without calibration against paleontological and paleoceanographic proxies^27^. Finally, the spatial modeling method is based on a deterministic approach strongly dependent upon an *a priori* definition of the carbonate factories. Today, carbonate factories and specific attributes can be observed at a platform scale. In the geological record however, the classification and characterization of carbonate factories is not straightforward as a result of incomplete preservation of geological parameters combined with paleobiological and paleoenvironmental uncertainties.

Prediction of the distribution of carbonate factories is a critical scientific challenge^8,25^ and has been undertaken many times in the last decades^15,27,29–32^. Since no numerical spatial model, accounting for all types of shallow-water marine carbonates, even existed in the Modern, this challenge seems even greater for past geological times. The carbonate factory concept^28^ and associated carbonate factory functions defined in this study represents a powerful predictive tool in formalizing relationships between carbonate sedimentology and oceanography^8,16,17^. Our spatial model constitutes a stepping stone toward the prediction of the distribution of different carbonate factories through space and time, on the basis of simple physical parameters. In addition, the carbonate factory concept provides geological information since carbonate factories are related to specific stratigraphic architectures, depositional profiles and facies associations^8,22^. The four carbonate factories studied here can be adapted and used in past geologic times, as demonstrated during Cretaceous times^27,28^. The carbonate factory functions could also be adapted to predict carbonate factories in the near future, which also represents a major interest in the context of ongoing climate change. Although only four environmental parameters are used here (i.e., SSS, SST, marine productivity and bathymetry), the modeling framework that we developed offers the possibility to consider additional parameters such *p*CO_2_, sea level, saturation or alkalinity. Such considerations would permit to work on a quantitative assessment of the carbonate production and thus to constitute a powerful tool to increase our understanding of the behavior of the global carbon cycle facing global climate change.

## Conclusion

This study presents a global predictive model of modern shallow-water marine carbonates based on carbonate factory functions that couple carbonate factories, bathymetry and measured oceanographic parameters, specifically seasonal SSS, SST and primary productivity. The four considered carbonate factories, biochemical, photozoan-T, photo-C and heterozoan-C factories, are characterized by specific oceanographic conditions and correspond to different ecological niches whose extent can be predicted. The biochemical, photozoan-T and photo-C factories are exclusive regarding the SST range and seasonality.

The carbonate factory functions simulate the carbonate factory distribution with an 82% fit to the observations. Carbonate factories do not overlap when defined at the platform scale, apart from the heterozoan-C factory, which is widespread and opportunistic, and is simulated in areas where other factories are inhibited. Terrigenous deposits are used as a spatial limitation to carbonate development. Uncertainties in the mapping of terrigenous material represent more than 57% of model anomalies. In particular, the poorly constrained extent of terrigenous deposits on large epicontinental shelves (e.g., Sunda Shelf and South China Sea) hampers finer-scale prediction of carbonate factories over such platforms. The spatial modeling method shows that a limited number of key historical oceanographic parameters (i.e., SST, SSS and marine productivity) provides a simple yet powerful tool to predict global distributions of shallow-water carbonate platforms. This method, which can be calibrated for past geological times and coupled with paleoceanographic models^27,28^, thus opens opportunities for the prediction of carbonate factories throughout past geological times or even future decades.

### Methods and data

#### Data

The observation map of worldwide carbonate distribution (Fig. 1) was built using more than 200 documents (published papers, maps and databases) on coral reefs and carbonate sediments. Tropical carbonates were first mapped from Wells (1988)^66–68^. Mapping of the carbonates of the Persian Gulf is based on Riegl *et al*. (2010 – see their Fig. 4.4)^33^. The distribution of the Mediterranean Sea coralligenous deposits mainly comes from Giakoumi *et al*.^69^. Heterozoan carbonates are referenced in areas where they represent >50% of shelfal sediments (as they are often mixed with terrigenous sediments). The database aims at referencing every modern carbonate sediment but it might not be completely exhaustive. Uncertainties include areas such as Southern South America, South Madagascar and the west coast of the USA that lack data (i.e., either publications about sedimentology or data dealing with carbonate sedimentology were not found). For every area where carbonate sediments are identified, the whole platform is mapped. The extent of platforms was assigned to a pre-defined water-depth interval, that is 0–100 m for tropical or photozoan carbonates and 0–200 m for heterozoan carbonates (see Supplementary Fig. S5).

The parameter maps of SST, SSS and oceanic primary productivity for both winter and summer seasons were obtained from remote-sensing data of AquaMODIS satellite^70,71^ (see Table 2 and Supplementary Fig. S6). The oceanic productivity data correspond to measurements of absorption due to phytoplankton (m^−1^). SST and oceanic productivity datasets are available at a resolution of 0.08°. Because the Aquamodis dataset of SSS shows a resolution of 1° and lacks data along continental coasts, SSS maps come from an interpolated map that was calculated from Aquarius data^72^. Salinities are classified into eight categories: 1-30–31.5 psu; 2 - 31.5–33 psu; 3 - 33–34 psu; 4 - 34–35 psu; 5 - 35–35.5 psu; 6 - 35.5–36.5 psu; 7 - 36.5–37.5 psu; 8 - 37.5 psu. The bathymetric map used in the model comes from the General Bathymetric Chart of the Ocean (GEBCO) 2014 release^73^.

**Table 2:** Characteristics of environmental parameter data.

Parameter	Temperature (SST)		Salinity (SSS)		Oceanic primary productivity		Bathymetry
	Summer	Winter	Summer	Winter	Summer	Winter	
Source	AquaMODIS SST (11 µ nighttime)		NASA scientific visualization studio. Flatmap based on the Aquarius data		AquaMODIS absorption due to phytoplankton at 443 nm, GIOP model		GEBCO_2014^73^
Period	2002–2014	2002–2015	1/08/2012	1/01/2012	2002–2015	2002–2015	—
Format	HDF		Image Raster		NetCDF		GRID
Initial resolution	9 km (~0.083°)		~5 km (~0.045°)		0.083°		900 m (~0.008°)
Final resolution	9 km (0.083°)						

A map of terrigenous sediments (Fig. 3) is used in the modeling process as a mask that excludes areas where carbonate sedimentation is inhibited or overwhelmed. The map of shelfal terrigenous sediments was developed using (i) remote-sensing images (Google Earth) and (ii) maps of external factors that control the occurrence and abundance of terrigenous input (i.e., the major rivers, drainage basin areas, topography and climate^51^). The combination of these four parameters provides an accurate worldwide distribution of areas where deposits are dominated by terrigenous sediments and thus where carbonate factories are excluded. Two categories of terrigenous inputs are defined: (i) “high terrigenous” that inhibits every carbonate factory and (ii) “low terrigenous” that does not inhibit the heterozoan-C factory occurring in mixed environments. The areal extent of terrigenous deposits could not be quantified here. Terrigenous areas were mapped along the shelves based on bathymetry. Under warm and humid climates (e.g., Southeast Asia), terrigenous deposits are widespread (also cf. “equatorial carbonates”^47^). Run-off from continent contains a high quantity of fine-grained sediments, due to high rainfall, which limits the growth of reefs. Nevertheless, seasonal longshore currents redistribute sediments along the coasts allowing luxurious carbonate production in zones where topography and/or currents limit terrigenous sediment deposition^74^ (i.e., a topographic feature, swept clean of terrigenous muds, could support suspension feeding biota). Remote-sensing-based (Google Earth) mapping of terrigenous sediments and plumes reveals this binary system; shelves are either flooded by terrigenous sediments or covered by reefs in protected areas. This type of mixed environments, where terrigenous and carbonate sediments alternate, is typical of humid areas^72^. In dry areas (e.g., in the Red Sea), terrigenous can also be present, but due to the coarseness of sediments and the periodicity of terrigenous input which is limited to rare episodic and short events, terrigenous fluxes do not impact carbonate development^72^.”

#### Statistical analyses and modeling process

Environmental parameter maps and the global map of carbonate factory distribution are superimposed in the GIS software. Oceanographic parameter values are extracted for each season from each cell in which carbonates are mapped. The purpose of the statistical study was to determine distributions and threshold values for each parameter and season for each carbonate factory (i.e., descriptive statistics). The parameter distributions and thresholds are used to calibrate the carbonate factory functions (see Supplementary Fig. S7). The functions combine the favorable oceanographic parameter values to define the oceanic zones that are favorable to the development of each carbonate factory.

The functions were defined using the analytic hierarchy process^75^ (AHP; see Supplementary Fig. S8) and fuzzy logic methods^76^, used in various scientific domains such as landslide assessment^77,78^ or gold exploration^79^:

1 $${\rm{F}}({\rm{carb}})=[{\rm{a}}\,{\rm{f}}(z)+{\rm{b}}\,{\rm{f}}(SST)+{\rm{c}}\,{\rm{f}}(SSS)+{\rm{d}}\,{\rm{f}}(P)]-[{\rm{terr}}]$$

where: *z* – Bathymetry; *SST* - Sea-surface temperature; *SSS* - Sea-surface salinity; *P* - Oceanic productivity (absorption due to phytoplankton); terr - Terrigenous deposits; f(*x*) - Susceptibility of occurrence for each environmental parameter *x*; a + b + c + d = 1 - inter-parameter weighting factors; F(carb) - Susceptibility of occurrence of the considered carbonate factory.

The f(*x*) functions represent the susceptibility of occurrence of carbonates depending on the environmental parameter *x*. The f(*x*) functions permit to normalize the values of the considered parameter between 0 and 1 (0 - complete inhibition of carbonate occurrences, 1 - completely favorable parameter value for carbonates to occur). The functions are designed using the results of the statistical study and the fuzzy logic method (see Supplementary Fig. S7). Depending on the shape of the distribution, one of these three types of functions is chosen:

2 $$\mathrm{Gaussian}:\,f(x)=\exp (\,-\,S\,{(x-MP)}^{2})$$

3 $${\rm{Near}}\,\mathrm{Gaussian}:\,f(x)=\frac{1}{1+(S\,{(x-MP)}^{2})}$$

4 $${\rm{Sigmoid}}\,({\rm{large}}):f(x)=\frac{1}{1+{(\frac{x}{MP})}^{-S}}$$

where MP is the Mid-Point, S is the Spread and *x* is the environmental parameter value.

The large sigmoïd function is used to normalize parameters when the considered factory shows an affinity for the highest parameter values (i.e., presence of a minimum threshold, but no maximum threshold). The gaussian and near-gaussian functions are used to normalize parameters when the factory shows an affinity for intermediate parameter values (i.e., presence of both minimum and maximum thresholds). The gaussian function corresponds to a normal distribution whereas the near gaussian function corresponds to a standard normal distribution (i.e., more weight is given to side values than in the gaussian function). We assume that gaussian and near gaussian functions represent the most pertinent choices to normalize parameters between two threshold values, even if the distributions obtained from the data sometimes show an asymmetric distribution. This choice was motivated by the fact that the observed asymmetry can be driven by some bias (due to sampling, geographic configuration, remote-sensing measurement, etc.), that are specific to the Modern carbonate distribution and environmental conditions. The normalization by a gaussian distribution ensures a more theoretical frame, more easily transferable to another dataset (for past geological times for instance).

The mathematical parameters of the functions (i.e., MP and S), are defined using the threshold values and/or the mode. For gaussian and near-gaussian functions, the Mid-Point is either the mean of the two threshold values or the mode of the descriptive statistics when only one threshold value is available; the spread is determined such as f(threshold value) is equal to 0.5. For Sigmoid functions, the Mid-Point is the threshold value itself; the spread is based on the descriptive statistics (mode and absolute minimum). S is determined by iterations and sensitivity tests to fit (i) the shape of the data distribution and (ii) the observation map.

The inter-parameter weighting factors represent the relative contribution of a parameter compared to other parameters. A higher weight is given to parameters having a stronger impact on carbonate occurrences. The weights are defined considering the statistical study and using pair-wise comparison according to the hierarchy of constraints that come from bibliography^9,22,23,26,33,49,80–82^ (Table 3; see Supplementary Table S9). Sensitivity tests are then conducted by comparing the modelled and observation maps in order to iteratively adjust the weighting factors.

**Table 3:** Bibliographic data of the driving environmental parameters of the carbonate factories.

Factory	Water depths (production + deposition)	Light-related depth of production	Temperature (SST)^21,33,46,62,64^	Salinity (SSS)^21,33,49,80,81^	Productivity (Absorption due to phytoplankton m^−1^)^23^
Biochemical	Few tens of meters; Maximum in the first few meters	Independent	>30 °C	>40 psu	Independent
Photozoan-T	0–100 m; maximum production: 0–15 m	Euphotic to mesophotic	Thresholds: 18 °C – 36 °C Optimum: 25–29 °C	Thresholds: 22–40 psu Optimum: 25–35 psu	Oligotrophic <0.4 m^−1^
Photo-C	0–200 m; maximum production: 0–120 m	Meso- to oligophotic	10–25 °C	35–38 psu	Oligo- to mesotrophic 0.4–1
Heterozoan-C	0–200 m	Independent	Independent	Independent	Meso- to eutrophic 1–10

The types of curves and associated parameters (Mid-Point and Spread) are detailed in the Table 1, as are weighting factors. For the bathymetry, a curve was also defined for each factory, only effective above 100 meters (200 for the heterozoan factory) as we used a mask to hide areas for deeper bathymetry values. Nevertheless, as the weight given to bathymetry is very low, no trend in carbonate distribution appeared to be controlled by the bathymetry curve. The curves, parameters and weighting factors for the bathymetry are available in Supplementary Table S10.

Two masks are used to the global modelled maps that constrain the zones of carbonate factory modeling. The first mask is the map of terrigenous deposits that excludes areas of high terrigenous sediment input. The second mask corresponds to deep bathymetries that limit the model runs to shallow-water platforms.

Once the f(*x*) functions and the weighting factors are determined, the final calculation of the F(carb) function provides a map of susceptibility of occurrence of shallow-water carbonates with values between 0 and 1. The susceptibility threshold is determined empirically by iteratively comparing the model output and the observations. In the model, carbonate factories are shown to occur only in cells displaying susceptibilities above the susceptibility threshold.

## Supplementary information

Supplementary informations

## Data Availability

Data used for this study are available upon request to the corresponding author.

## References

[1] Knowlton, N. *et al*. Coral reef biodiversity in *Life in the World’s Oceans: Diversity, Distribution, Abundance* (ed. Wiley-Blackwell, Oxford, UK) 65–78 (2010).

[2] Jones NS, Ridgwell A, Hendy EJ (2015). Evaluation of coral reef carbonate production models at a global scale. Biogeosciences.

[3] Falkowski P (2000). The global carbon cycle: a test of our knowledge of earth as a system. Science.

[4] Wilson, J. L. *Carbonate Facies in Geologic History* (ed. Springer–Verlag, New York) 1–471 (1975).

[5] Tucker, M. E. & Wright, V. P. *Carbonate sedimentology* (ed. Blackwell, Oxford) 1–496 (1990).

[6] Whalen MT (1995). Barred basins: a model for eastern ocean basin carbonate platforms. Geology.

[7] James, N. P. & Jones, B. G. The cool-water neritic realm in *Origin of Carbonate Rocks* (ed. AGU, John Wiley & Sons) 135–149 (2015).

[8] James, N. P. The cool-water carbonate depositional realm in *Cool-water carbonates*, *SEPM Spec. Publ*. **56** (ed. James N. P. & Clarke J. A. D.) 1–20 (1997).

[9] Schlager W (2005). *Carbonate Sedimentology and Sequence Stratigraphy*. SEPM Concepts in Sedimentology and Paleontology.

[10] Pomar L, Hallock P (2008). Carbonate factories: a conundrum in sedimentary geology. Earth Sci. Rev..

[11] Lees A, Buller AT (1972). Modern temperate-water and warm-water shelf carbonate sediments contrasted. Mar. Geol..

[12] Bosence D (2005). A genetic classification of carbonate platforms based on their basinal and tectonic settings in the Cenozoic. Sediment. Geol..

[13] Pomar LWC (2001). Types of carbonate platforms - a genetic approach. Basin Res..

[14] Pomar. L. W. C. & Kendall, C. G. Ecological Accommodation; A Key to the Interpretation of Carbonate Platform Architecture Variability. *Abstract AAPG Annual Convention, San Antonio, TX*, 20–23 (2008).

[15] Lees A (1975). Possible influence of salinity and temperature on modern shelf carbonate sedimentation. Mar. Geol..

[16] Schlager, W. Sedimentation rates and growth potential of tropical, cool-water and mud-mound carbonate factories in *Carbonate platform systems: components and interactions*. *Geol. Soc. Lond. Spec. Publ*. **178** (ed. Insalaco E., Skelton P. & Palmer T. J.) 217–227 (2000).

[17] Schlager W (2003). Benthic carbonate factories of the Phanerozoic. J. Earth. Sci..

[18] Nelson CS (1988). An introductory perspective on non-tropical shelf carbonates. Sediment. Geol..

[19] Henrich R (1995). Controls on modern carbonate sedimentation on warm temperate to Arctic coasts, shelves and seamounts in the northern hemisphere; implications for fossil counterparts. Facies.

[20] Jansa L (1996). Modern Carbonates: Tropical, Temperate, Polar - Introduction to Sedimentology and Geochemistry. Sediment. Geol..

[21] Betzler C, Brachert T, Nebelsick JH (1997). The warm temperate carbonate province – a review of the facies, zonations, and delimitations. Cour. Forsch. Inst. Senckenberg.

[22] Michel J, Borgomano J, Reijmer JJG (2018). Heterozoan carbonates: When, where and why? A synthesis on parameters controlling carbonate production and occurrences. Earth Sci. Rev..

[23] Hallock, P. Coral reefs, carbonate sediments, nutrients, and global change in *History and Sedimentology of Ancient Reef Systems* (ed. Stanley, G. D., The Springer, Boston, MA) 387–427 (2001).

[24] Kiessling W, Flügel E, Golonka J (2003). Patterns of Phanerozoic carbonate platform sedimentation. Lethaia.

[25] Markello JR, Koepnick RB, Waite LE, Collins JF (2007). The carbonate analogs through time (CATT) hypothesis and the clobal atlas of carbonate fields – A systemic and predictive look at phanerozoic carbonate systems in *Controls on Carbonate Platform and Reef*. Development, SEPM Spec. Publ..

[26] Kleypas JA, McManus JW, Menez LAB (1999). Environmental limits to coral reef development: where do we draw the line?. Am. Zool..

[27] Pohl A (2019). Quantifying the paleogeographic driver of Cretaceous carbonate platform development using paleoecological niche modeling. Pal. Pal. Pal..

[28] Michel J (2019). Marine carbonate factories: a global model of carbonate platform distribution. Int. J. Earth Sci. (Geol Rundsch).

[29] Lough JM (2008). Coral calcification from skeletal records revisited. Mar. Ecol. Prog. Ser..

[30] Kleypas J (1997). Modeled estimates of global reef habitat and carbonate production since the last glacial maximum. Paleoceanography.

[31] Anthony KRN, Kleypas JA, Gattuso JP (2011). Coral reefs modify their seawater carbon chemistry – case study from a barrier reef (Moorea, French Polynesia). Global Change Biol..

[32] Martin, C.S. *et al*. Coralligenous and maerl habitats: predictive modeling to identify their spatial distributions across the Mediterranean Sea. *Sci. Rep*. **4**, 5073; 10.1038/srep05073 (2014).

[33] Riegl, B., Poiriez, A., Janson, X. & Bergman, K. L. The Gulf: Facies belts, physical, chemical, and biological parameters of sedimentation on a carbonate ramp in *Carbonate Depositional Systems: Assessing Dimensions and Controlling Parameters - The Bahamas, Belize and the Persian/Arabian Gulf* (ed. Westphal, H., Riegl, B. & Eberli, G. P., Springer, Dordrecht Heidelberg London New York) 145–213 (2010).

[34] Purser BH (1969). Syn-sedimentary marine lithification of Middle Jurassic limestones in the Paris basin. Sedimentology.

[35] Riegl B (1999). Corals in a non-reef setting in the southern Arabian Gulf (Dubai, UAE): fauna and community structure in response to recurring mass mortality. Coral Reefs.

[36] Riegl B (2001). Inhibition of reef framework by frequent disturbance: examples from the Arabian Gulf, South Africa, and the Cayman Islands. Pal. Pal. Pal..

[37] Geister J (1983). Holozäne westindische Korallenriffe: Geomorphologie, Oëkologie und Fazies. Facies.

[38] Dupraz C (2009). Processes of carbonate precipitation in modern microbial mats. Earth Sci. Rev..

[39] Bouton A (2016). External controls on the distribution, fabrics and mineralization of modern microbial mats in a coastal hypersaline lagoon, Cayo Coco (Cuba). Sedimentology.

[40] Geiger, R. & Überarbeitete Neuausgabe von Geiger, R.: Köppen-Geiger / Klima der Erde. (Wandkarte 1:16 Mill.) – Klett-Perthes, Gotha (1961).

[41] Peel, M. C., Finlayson, B. L. & Mcmahon, T. A. Updated world map of the Köppen-Geiger climate classification. *Hydrol. Earth. Syst. Sci. Discussions* **11**, 1633–1644 (2007), https://hal.archives-ouvertes.fr/hal-00305098 (2019).

[42] Pérès, J. M. & Picard, J. Nouveau manuel de bionomie benthique de la Mer Méditerranée in *Recueil de la Station Marine d’Endoume* **31**, 5–137 (1964), http://paleopolis.rediris.es/BrachNet/REF/Download/Manuel.html (2019).

[43] Pedley, H. M. & Carannante, G. Cool-water carbonate ramps: a review in *Cool-water Carbonates: Depositional Systems and Palaeoenvironmental Controls, Geol. Soc. Lond., Spec. Publ*. **255** (ed. Pedley, H. M. & Carannante, G.) 1–9 (2006).

[44] Wood R (1993). Nutrients, predation and the history of reef-building. Palaios.

[45] Westphal H, Halfar J, Freiwald A (2010). Heterozoan carbonates in subtropical to tropical settings in the present and past. Int. J. Earth Sciences.

[46] Pedley HM, Carannante G (2006). Cool-water carbonates: depositional systems and paleoenvironmental controls. Geol. Soc. Lond., Spec. Publ..

[47] Halfar J, Godinez-Orta L, Mutti M, Valdez-Holguin JE, Borges JM (2004). Nutrient and temperature controls on modern carbonate production: an example from the Gulf of California, Mexico. Geology.

[48] James NP, Bone Y, Collins LB, Kurtis Kyser T (2001). Surficial sediments of the Great Australian Bight: facies dynamics and oceanography on a vast cool-water carbonate shelf. J. Sediment. Res..

[49] James, N. P. & Bone, Y. *Neritic Carbonate Sediments in a Temperate Realm* (ed. Springer, Dordrecht Heidelberg London New York) 1-254 (2011).

[50] Jokiel PL (1978). Effects of water motion on reef corals. J. Exp. Mar. Biol. Ecol..

[51] Dennison WC, Barnes DJ (1987). Effect of water motion on coral photosynthesis and calcification. J. Exp. Mar. Biol. Ecol..

[52] Pomar L, Baceta JI, Hallock P, Mateu-Vicens G, Basso D (2017). Reef building and carbonate production modes in the west-central Tethys during the Cenozoic. Mar. Petrol. Geol..

[53] Wilson MEJ (2012). Equatorial carbonates: an earth systems approach. Sedimentology.

[54] Milliman, J. D. & Farnsworth, K. L. *River Discharge to the Coastal Ocean – A Global Synthesis* (ed. Cambridge University Press) 1–394 (2011).

[55] Sheppard, C., Price, A. & Roberts, C. *Marine Ecology of the Arabian Region: Patterns and Processes in Extreme Tropical Environments* (ed. Academic Press, London) 1–359 (1992).

[56] Wasserman, H. N. A Global View of Coral Reef Cementation as a Function of Seawater Aragonite Saturation States. *Undergraduate Honors Theses* **722** (2011).

[57] James NP, Collins LB, Bone Y, Hallock P (1999). Subtropical carbonates in a temperate realm: modern sediments on the southwest Australian shelf. J. Sediment. Res..

[58] Jiang LQ (2015). Climatological distribution of aragonite saturation state in the global oceans. Glob. Biogeochem. Cycl..

[59] Jiang ZP, Tyrrell T, Hydes DJ, Dai M, Hartman SE (2014). Variability of alkalinity and the alkalinity-salinity relationship in the tropical and subtropical surface ocean. Glob. Biogeochem. Cycl..

[60] Carter BR, Toggweiler JR, Key RM, Sarmiento JL (2014). Processes determining the marine alkalinity and calcium carbonate saturation state distributions. Biogeosciences.

[61] Arp G, Reimer A, Reitner J (1999). Calcification in cyanobacterial biofilms of alkaline salt lakes. Eur. J. Sediment. Res..

[62] Arp G, Reimer A, Reitner J (2003). Microbialite formation in seawater of increased alkalinity, Satonda Crater Lake, Indonesia. Eur. J. Phycol..

[63] Perry CT, Larcombe P (2003). Marginal and non-reef building coral environments. Coral Reefs.

[64] Goddéris Y (2017). Onset and ending of the late Palaeozoic ice age triggered by tectonically paced rock weathering. Nat. Geosci..

[65] Ridgwell A, Zeebe RE (2005). The role of the global carbonate cycle in the regulation and evolution of the Earth system. Earth. Planet. Sc. Lett..

[66] Wells, S. M. *Coral Reefs of the World. Volume 1: Atlantic and Eastern Pacific* (ed. UNEP Regional Seas Directories and Bibliographies. IUCN, Gland, Switzerland and Cambridge, U.K./UNEP, Nairobi, Kenya) 1–436 (1988), https://www.coral.noaa.gov/resources/maps.html (2019).

[67] Wells, S. M. *Coral Reefs of the World. Volume 2: Indian Ocean, Red Sea and Gulf*. (ed. UNEP Regional Seas Directories and Bibliographies. IUCN, Gland, Switzerland and Cambridge, U.K./UNEP, Nairobi, Kenya 1–389 (1988), https://www.coral.noaa.gov/resources/maps.html (2019).

[68] Wells, S. M. *Coral Reefs of the World. Volume 3: Central and Western Pacific*. (ed. UNEP Regional Seas Directories and Bibliographies. IUCN, Gland, Switzerland and Cambridge, U.K./UNEP, Nairobi, Kenya) 1–329 (1988), https://www.coral.noaa.gov/resources/maps.html (2019).

[69] Giakoumi, S. *et al*. Ecoregion-Based Conservation Planning in the Mediterranean: Dealing with Large-Scale Heterogeneity. *PLOS One* **8**, 10.1371/journal.pone.0076449 (2013). 10.1371/journal.pone.0076449 PMC3796553 24155901

[70] NASA Goddard Space Flight Center, Ocean Ecology Laboratory, Ocean Biology Processing Group. Moderate-resolution Imaging Spectroradiometer (MODIS) Aqua Sea Surface Temperature Data; 2014 Reprocessing. NASA OB.DAAC, Greenbelt, MD, USA. data, 10.5067/AQUA/MODIS/L3B/SST/2014., https://oceancolor.gsfc.nasa.gov (2015).

[71] NASA Goddard Space Flight Center, Ocean Ecology Laboratory, Ocean Biology Processing Group. Moderate-resolution Imaging Spectroradiometer (MODIS) Binned Remote-Sensing Reflectance. Data; 2014 Reprocessing. NASA OB.DAAC, Greenbelt, MD, USA, 10.5067/AQUA/MODIS/L3B/RRS/2014, https://oceancolor.gsfc.nasa.gov (2015).

[72] NASA’s Scientific Visualization Studio. Aquarius/Microwave Radiometer/Sea Surface Salinity, http://svs.gsfc.nasa.gov/4353 (2015).

[73] General Bathymetric Chart of the Oceans (GEBCO), GEBCO_2014 grid, version 20141103, https://www.gebco.net/data_and_products/gridded_bathymetry_data (2015).

[74] Friedman GM (1988). Case histories of coexisting reefs and terrigenous sediments: the Gulf of Elat (Red Sea), Java Sea, and Neogene basin of the Negev, Israël. Dev. Sedimentol..

[75] Saaty TL (1970). How to Make a Decision: The Analytic Hierarchy Process. Eur. J. Oper. Res..

[76] Zadeh LA (1965). Fuzzy sets. Inform. Control..

[77] Thiery Y, Maquaire O, Fressard M (2014). Application of expert rules in indirect approaches for landslide susceptibility assessment. Landslides.

[78] Yoshimatsu H, Abe S (2006). A review of landslide hazards in Japan and assessment of their susceptibility using an analytical hierarchic process (AHP) method. Landslides.

[79] Nouri R, Afzal P, Arian M, Jafari M, Feizi F (2013). Reconnaissance of copper and gold mineralization using analytical hierarchy process (AHP) in the rudbar 1:100,000 map sheet, Northwest Iran. J. Min. Metall. A..

[80] James NP, Bourque PA, Walker RG, James NP (1992). Reefs and mounds in *Facies Models - Response to Sea Level Change*. Geological Association of Canada Geotext.

[81] Couce E, Ridgwell A, Hendy EJ (2012). - Environmental controls on the global distribution of shallow-water coral reefs. J. Biogeogr..

[82] Ballesteros E (2006). Mediterranean coralligenous assemblages: a synthesis of present knowledge. Oceanogr. Mar. Biol..

