# Supplementary material for: Global distribution of modern shallow-water marine carbonate factories: a spatial model based on environmental parameters
Source: Sci Rep. 2019 Nov 11;9:16432. doi: 10.1038/s41598-019-52821-2 (PMC6848134; doi:10.1038/s41598-019-52821-2)
Supplement: Supplementary file 1 — Supplementary informations [file 41598_2019_52821_MOESM1_ESM.pdf]

Supplementary online materials for

Global distribution of modern shallow-water marine carbonate factories: a spatial model based on environmental parameters

Marie Laugié[1,2*], Julien Michel[1,2], Alexandre Pohl[1], Emmanuelle Poli[3], Jean Borgomano[1,3]

[1]Aix Marseille Univ, CNRS, IRD, INRA, Coll France, CEREGE, Aix-en-Provence, France

[2]MODIS Pau, 4 Rue Jules Ferry, Pau, France

[3]Total CSTJF, Avenue Larribeau, 64000 Pau, France

Correspondence to: M. Laugié (marielaugie@gmail.com)

Contents

- Supplementary Figures S1 to S3, S5 to S8
- Supplementary Tables S4 and S9, S10

Results: Modelling framework

Supplementary Figure S1: Global modelling framework: representation of the different steps of the modelling process, from the spatial analysis to the final simulated map of carbonate factory distribution, including the definition of carbonate factory functions. Input data are the SSS, the SST, the marine primary productivity maps for summer and winter seasons, and the bathymetry map. The final output is a global map showing the absence or presence of the four factories. The parameter maps of SST, SSS and oceanic primary productivity for both winter and summer seasons are obtained from remote-sensing data of AquaMODIS satellite (https://oceancolor.gsfc.nasa.gov/). The complete workflow, i.e., mapping, data processing, spatial statistics and modeling, is realized using the Environmental Systems Research Institute (ESRI) software ArcGIS (http://desktop.arcgis.com/fr/arcmap/ - v10.2.2).

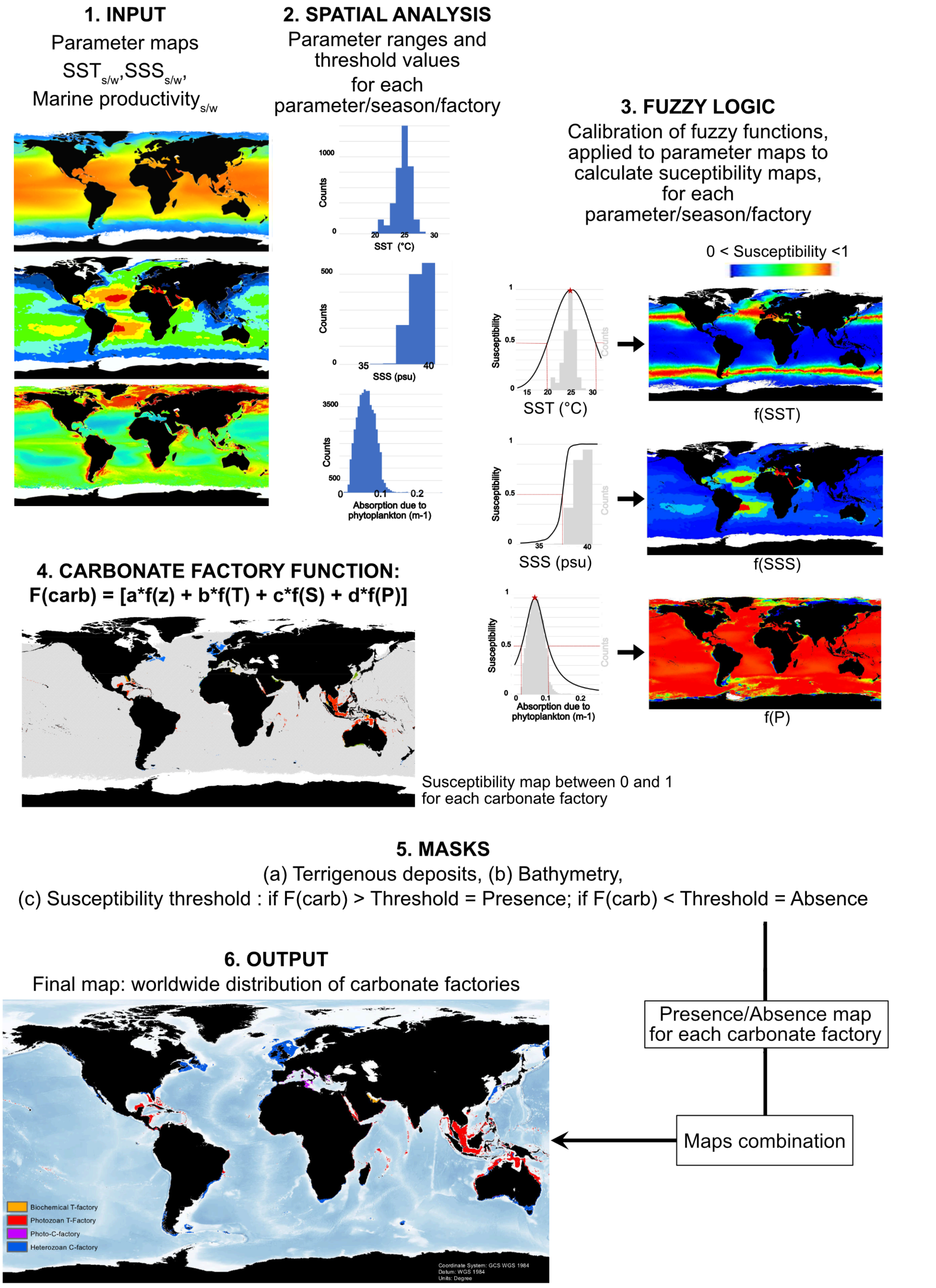

Results: Spatial analysis and carbonate factory definition

✦ The tropical zone: the photozoan-T factory

For the photozoan-T factory, temperatures have to rise above 24 °C, at least seasonally, to produce the carbonate factory, but it can persist if temperatures drop down to 18 °C. Below 18 °C, the photozoan-T factory is absent.

Supplementary Figure S2: Photozoan-T factory - production (> 24 °C) vs. persistence (> 18 °C). The south-west corner of Western Australia does not host the photozoan-T factory. This can be explained by the definition of two critical thresholds: (1) 24 °C as a minimum SST for carbonate production and (2) 18 °C as a minimum SST for the carbonate factory persistence. Thus, areas between 18 °C and 24 °C all year round do not allow the photozoan-T factory to occur. The parameter maps of SST for both winter and summer seasons are obtained from remote-sensing data of AquaMODIS satellite (https://oceancolor.gsfc.nasa.gov/), and classified using the Environmental Systems Research Institute (ESRI) software ArcGIS (http://desktop.arcgis.com/fr/arcmap/ - v10.2.2). The satellite imagery (right picture) was obtained from Google Maps (https://www.google.fr/maps/).

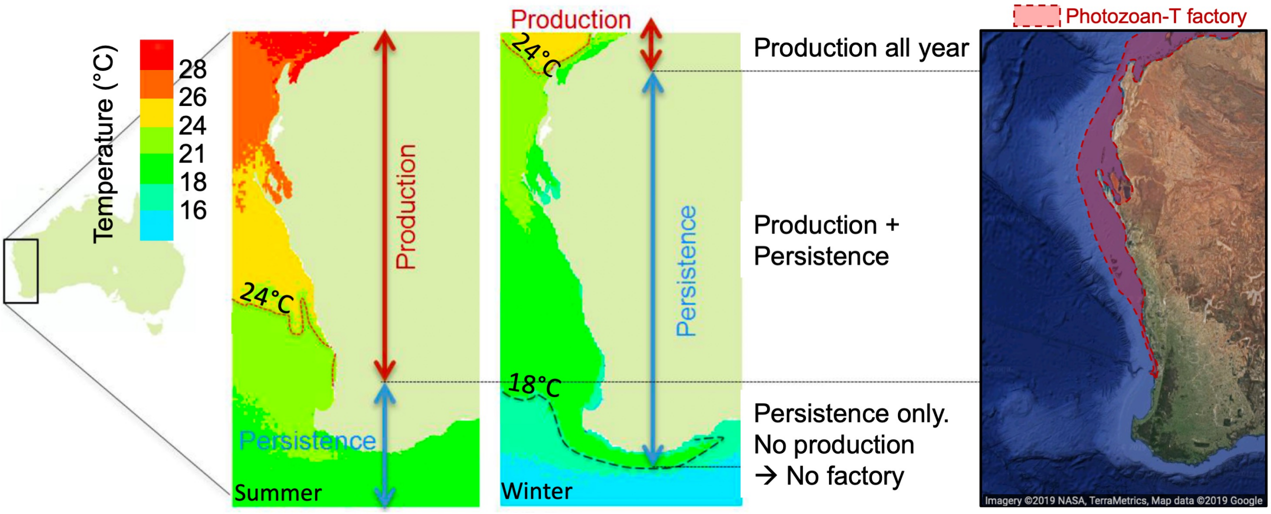

Results: From environmental parameters to the carbonate factory functions - Modeled distribution of carbonate factories

Supplementary Figure S3: Simulated modern carbonate factory distribution. The worldwide simulated map is obtained using the carbonate factory functions. Global trends are well reproduced. The main overestimations of the photozoan-T and heterozoan-C factories are observed on large epicontinental areas which are influenced by terrigenous deposits, e.g. Sunda, South-China and North Sea continental shelves.

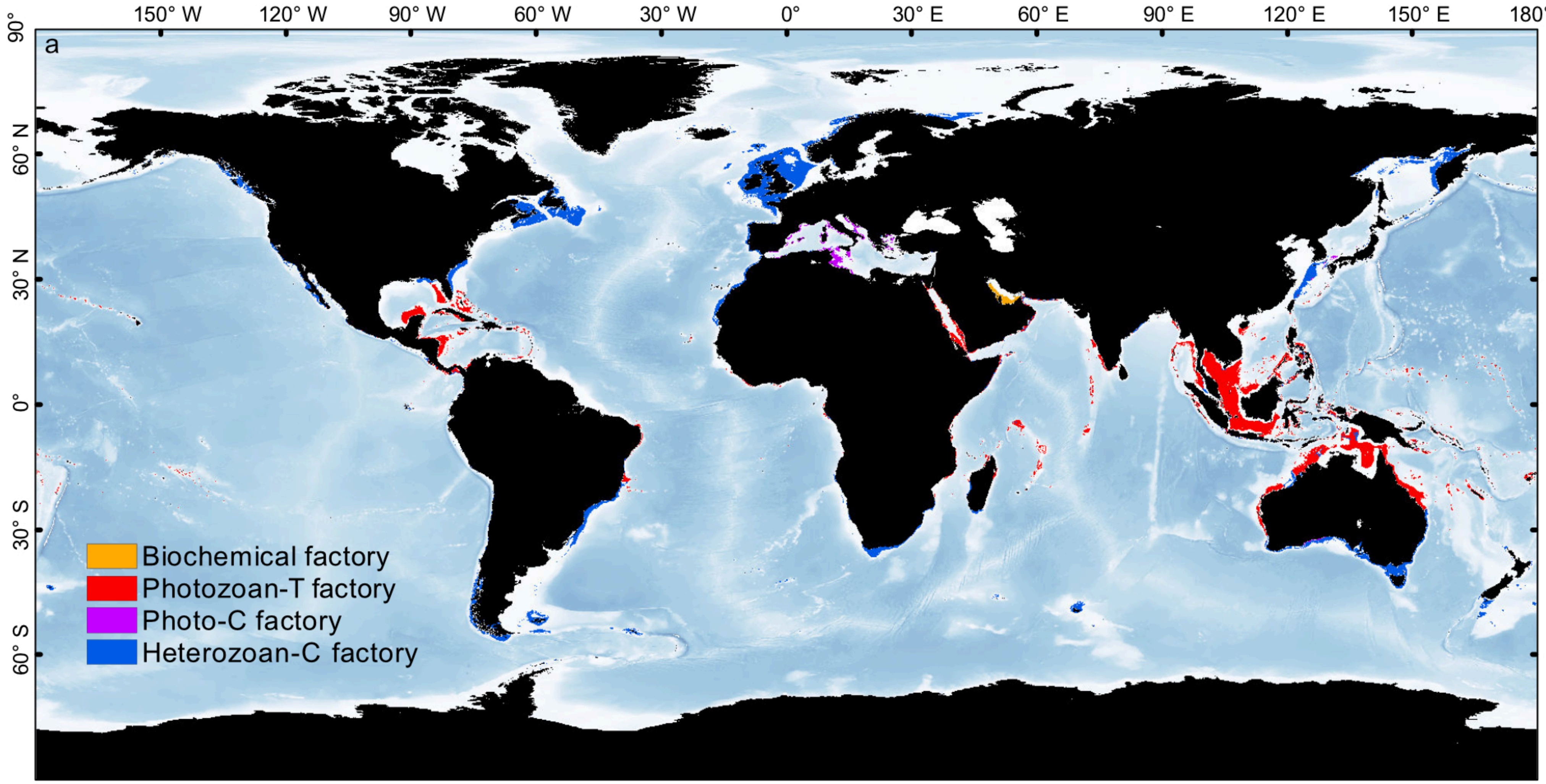

a
150° W
120° W
90° W
60° W
30° W
0°
30° E
60° E
90° E
120° E
150° E
180°
90°
60° N
30° N
0°
30° S
60° S
Biochemical factory
Photozoan-T factory
Photo-C factory
Heterozoan-C factory

Results: From environmental parameters to the carbonate factory functions - Model anomalies

Supplementary Table S4: Calculation of modelling anomalies. (a-d) Modelling anomalies for each carbonate factory. A success is counted when modelled and reference maps fit (absence or presence). An anomaly is counted when the reference and modelled maps disagree on the absence/presence of the considered factory. (e) The global anomaly corresponds to the sum of anomalies for each factory. Blue numbers represent success and red numbers represent anomalies (unit = number of cells).

a – Biochemical factory<br>Total success = 99.8 %		Modelled map	
		Absence	Presence
Reference map	Absence	71748	85
	Presence	41	1257

b – Photozoan-T factory<br>Total success = 65 %		Modelled map	
		Absence	Presence
Reference map	Absence	27818	25799
	Presence	1277	22482

c – Photo-C factory<br>Total success = 98.2 %		Modelled map	
		Absence	Presence
Reference map	Absence	113170	1485
	Presence	498	2706

d – Heterozoan-C factory<br>Total success = 70.4 %		Modelled map	
		Absence	Presence
Reference map	Absence	78574	38655
	Presence	7523	30684

e – TOTAL<br>Total success = 82 %		Modelled map	
		Absence	Presence
Reference map	Absence	286621	66024
	Presence	9069	57129

Data and Methods

1. Data

Supplementary Figure S5: Mapping method. The mapping was realized using (1) bibliography to localize the carbonates and (2) the bathymetry map to fix their extent between 0 - 100 meters (photozoan-T and biochemical factories) or 0 - 200 meters (photo-C and heterozoan C-factories).

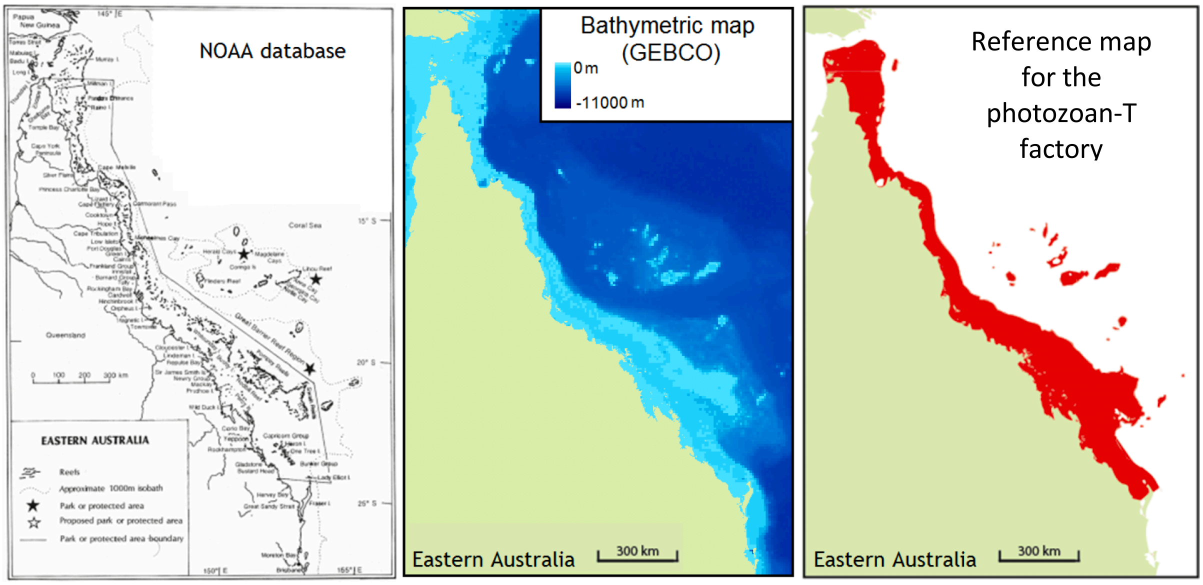

Supplementary Figure S6: Parameter maps. Maps used for the spatial analysis and modelling, from top to bottom: SST (°C), SSS (psu), absorption due to phytoplankton (m^{-1}) and bathymetry (m). Left column: summer and right column: winter. The parameter maps of SST, SSS and oceanic primary productivity for both winter and summer seasons are obtained from remote-sensing data of AquaMODIS satellite (https://oceancolor.gsfc.nasa.gov/).

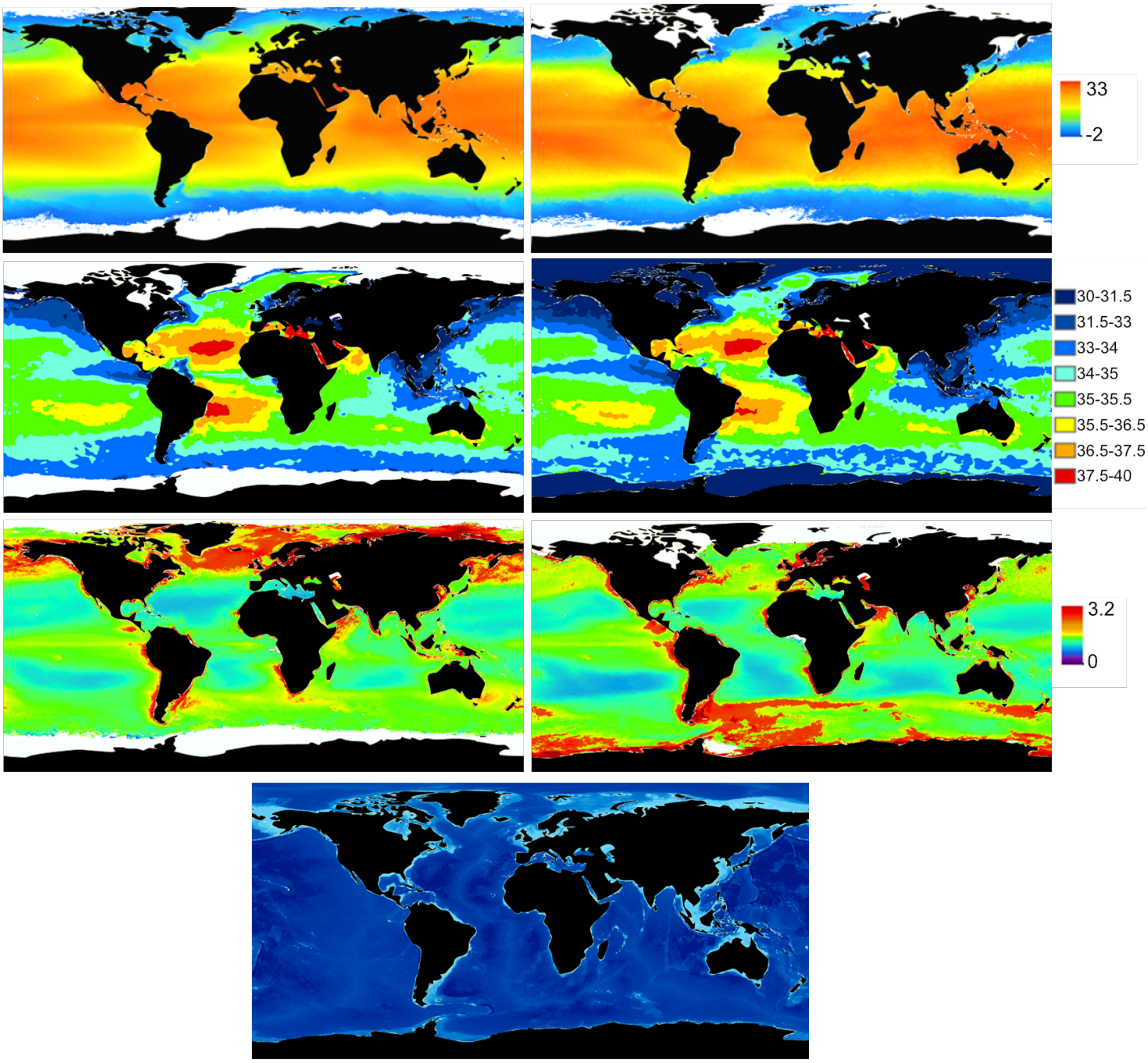

Supplementary Figure S7: Fuzzy function definition. (a) Choice of the type of function (gaussian, near-gaussian or sigmoïd). (b) Adjustment of spread (S) and mid-point (MP) for a sigmoïd case; MP = first percentile value; S = adjusted such as f(absolute minimum) = 0.05. (c) Adjustment of spread (S) and mid-point (MP) for gaussian and near-gaussian cases: MP = mean of minimum and maximum threshold values and S = adjusted such as f(minimum threshold) = f(maximum threshold) = 0.5.

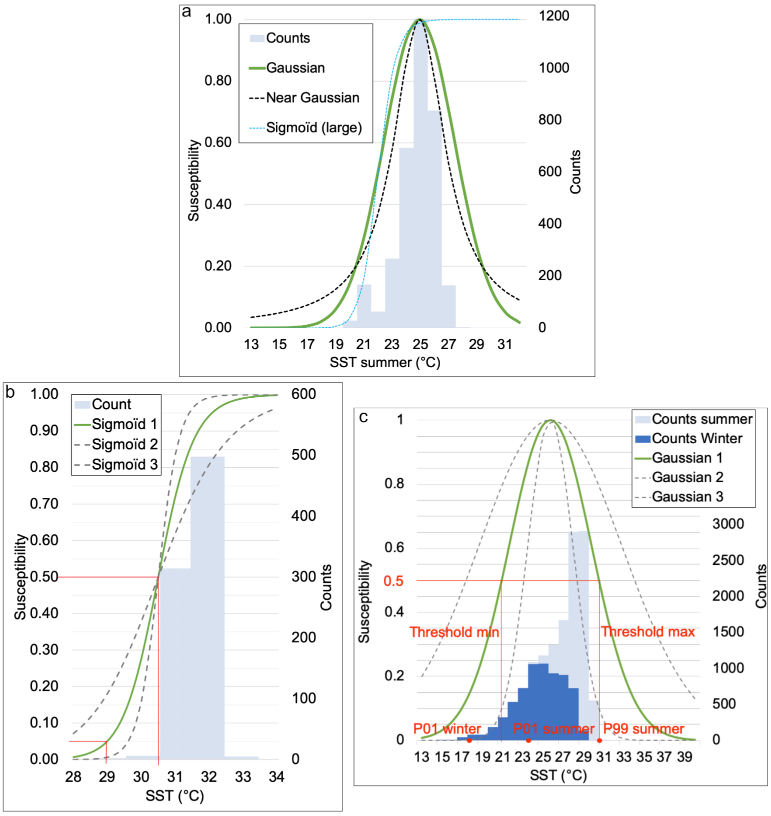

Supplementary Figure S8: AHP method. To achieve the "goal", determining criteria are hierarchized and a weight is assigned to each one by using pair-wise comparison. The sum of critera weighting factors (a+b+c+d) must be equal to 1, and for one specific criterion, the sum of sub-criteria weighting factors must also be equal to 1. In this study, the "goal" is the occurrence of the carbonate factory, the criteria are the bathymetry, SST, SSS and marine productivity and the sub-criteria are the seasons. For the seasons, weighting factors are set to 0.5 for both summer and winter. For parameter weighting factors, see the main text and Table S2.

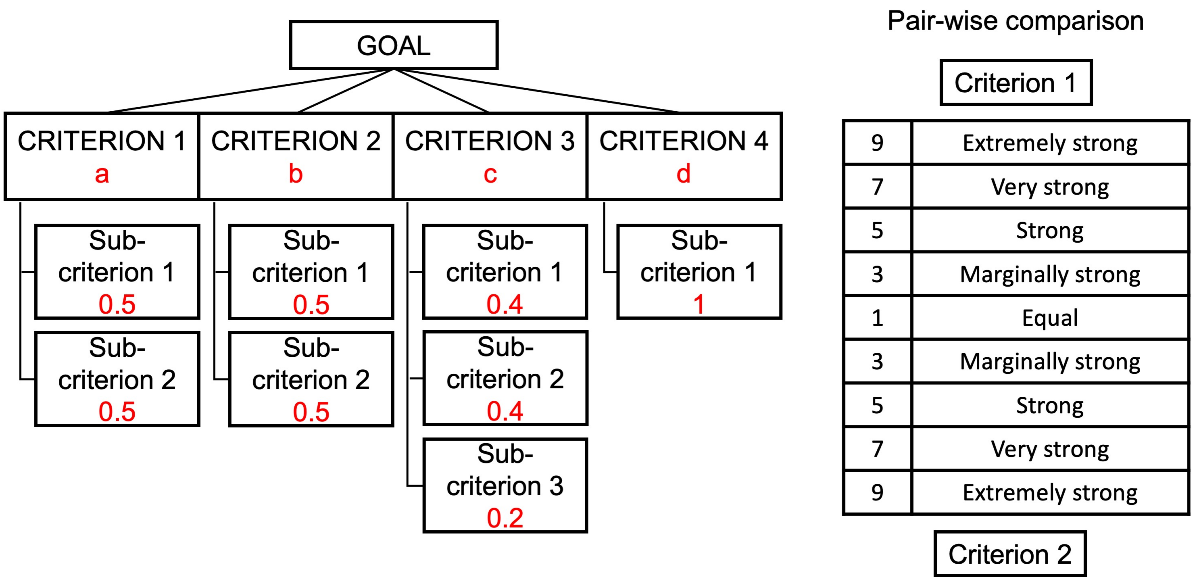

Supplementary Table S9: Pair-wise comparison matrix. Pair-wise comparison has been realized based on bibliography and results of the spatial analysis, and adjusted by iterations to obtain the best fit between modelled and reference maps. Bathymetry is always included in the weighting factor calculation but its role is considered as minor because it is only used as a mask. (a) Biochemical factory: temperature and salinity are identified as "equally important" parameters. (b) Photozoan-T factory: temperature importance is "extremely strong" compared to salinity, and "strong" compared to marine primary productivity. Marine primary productivity is "extremely strong" compared to salinity. (c) Photo-C factory: temperature importance is "very strong" compared to salinity, and "strong" compared to marine primary productivity. Marine primary productivity is "marginally strong" compared to salinity. (d) Heterozoan-C factory: marine primary productivity is the only considered parameter (apart from the bathymetry).

a – Biochemical factory	Temperature	Salinity	Bathymetry
Temperature		1	5
Salinity	1		5
Bathymetry	1/5	1/5	

b – Photozoan-T factory	Temperature	Salinity	Productivity	Bathymetry
Temperature		9	5	9
Salinity	1/9		1/9	3
Productivity	1/5	9		7
Bathymetry	1/9	1/3	1/7	

c – Photo-C factory	Temperature	Salinity	Productivity	Bathymetry
Temperature		7	5	9
Salinity	1/7		1/3	5
Productivity	1/5	3		7
Bathymetry	1/9	1/5	1/7	

d –Heterozoan-C factory	Productivity	Bathymetry
Productivity		9
Bathymetry	1/9	

Supplementary Table S10: Bathymetry parametrization. Types of functions and associated mathematical parameters and inter-parameter weighting factors for each carbonate factory.

Factory	Biochemical	Photozoan-T	Photo-C	Heterozoan-C
Type of function	"Sigmoid - Small"	"Gaussian"	"Sigmoid – Small"	"Gaussian Near"
Mid-Point	25	15	100	50
Spread	4.25	0.0011	5	0.0002
Weighting factor	0.1	0.4	0.4	0.1
